# Point Cloud Denoising and Feature Preservation: An Adaptive Kernel Approach Based on Local Density and Global Statistics

**DOI:** 10.3390/s24061718

**Published:** 2024-03-07

**Authors:** Lianchao Wang, Yijin Chen, Wenhui Song, Hanghang Xu

**Affiliations:** College of Geoscience and Surveying Engineering, China University of Mining and Technology (Beijing), Beijing 100083, China; y.j.chen@cumtb.edu.cn (Y.C.); bqt1800205061@student.cumtb.edu.cn (W.S.); bqt2000205068@student.cumtb.edu.cn (H.X.)

**Keywords:** point cloud, denoising, Bayesian estimation, adaptive kernel density

## Abstract

Noise removal is a critical stage in the preprocessing of point clouds, exerting a significant impact on subsequent processes such as point cloud classification, segmentation, feature extraction, and 3D reconstruction. The exploration of methods capable of adapting to and effectively handling the noise in point clouds from real-world outdoor scenes remains an open and practically significant issue. Addressing this issue, this study proposes an adaptive kernel approach based on local density and global statistics (AKA-LDGS). This method constructs the overall framework for point cloud denoising using Bayesian estimation theory. It dynamically sets the prior probabilities of real and noise points according to the spatial function relationship, which varies with the distance from the points to the center of the LiDAR. The probability density function (PDF) for real points is constructed using a multivariate Gaussian distribution, while the PDF for noise points is established using a data-driven, non-parametric adaptive kernel density estimation (KDE) approach. Experimental results demonstrate that this method can effectively remove noise from point clouds in real-world outdoor scenes while maintaining the overall structural features of the point cloud.

## 1. Introduction

Presently, the acquisition and processing of 3D data have become key technologies [1,2]. These technologies play a crucial role in various fields, including innovative basic surveying and mapping [3,4,5], the creation of high-precision maps for autonomous driving [6,7,8,9], geological landslide monitoring [10,11], and the preservation of cultural heritage [12,13,14,15,16]. Point cloud data, as a primary form of 3D data, are highly favored for their ability to precisely depict the physical space’s shape and structure. However, due to factors such as measurement errors and environmental interference, the original point cloud data often contain noise. This not only affects the quality of the data but also interferes with subsequent data processing and analysis. Therefore, developing effective denoising techniques is important for enhancing the utility value of point clouds.

Despite significant achievements in the field of point cloud denoising by related research [17], particularly in recent years with the infiltration of deep learning technology in the domain of point clouds, algorithms for point cloud denoising based on the deep learning approach have seen rapid development [18,19,20,21]. However, due to the inherent unstructured nature of point clouds and the fundamentally ill-posed nature of the point cloud denoising task, existing denoising algorithms still exhibit varying degrees of limitation [22]. Specifically, most current algorithms assess and demonstrate point cloud denoising effects primarily on CAD models [23,24]. This approach results in a lack of flexibility and universality for the denoising algorithms, leading to generally mediocre performance on point clouds scanned in real-world outdoor scenes. Moreover, existing algorithms often employ a step of adding predefined noise types to models before filtering out noise. For instance, they might disturb the input point cloud with Gaussian noise to generate a noisy model. This method of artificially simulating noise results in a somewhat homogenous noise environment for the denoising algorithms, reducing their effectiveness when confronted with complex noise that lacks a clear mathematical definition. Therefore, developing algorithms capable of handling point cloud denoising in real-world outdoor scenes becomes particularly prominent and important. In this context, the theory and application of discrete orthogonal moments offer a new perspective and tools for point cloud denoising. Specifically, the application of Krawtchouk moments and their variants in image reconstruction [25] and image analysis [26], as well as the use of Charlier–Meixner moments in image classification [27] and Meixner moments in the fast computation of inverse transforms [28], demonstrates the potential of discrete orthogonal moments in handling image and point cloud data; these studies provide new insights for point cloud denoising.

This study introduces a novel point cloud denoising method that integrates probabilistic statistics with spatial analysis. This method not only considers the spatial distribution characteristics of point clouds but also incorporates probabilistic models to differentiate between noise points and real points more accurately. By combining KD-tree spatial indexing techniques with kernel density estimation, this method can effectively process large-scale point clouds, achieving high denoising precision while preserving environmental features. The noise in point clouds mainly originates from two aspects: random errors during the data acquisition process and systematic errors caused by object surface characteristics or environmental factors. Traditional denoising methods typically employ neighborhood analysis techniques [29], such as radius filtering, median filtering, and statistical filtering. These methods are effective in dealing with random noise, but often fall short when addressing systematic errors. In contrast, the method proposed in this study dynamically adjusts prior probabilities and likelihood functions, accurately reflecting the spatial distribution and statistical characteristics of points. This method not only effectively removes noise but also significantly reduces systematic errors, achieving a favorable balance between noise removal and the preservation of environmental features.

Furthermore, this study pays special attention to the scale issue of point clouds. In large-scale outdoor point clouds, there can be significant differences in point density across different areas [30,31,32,33], posing higher demands on denoising algorithms. This method introduces a combination of global and local bandwidths, along with Silverman’s rule of thumb and kernel density estimation, enabling it to adapt to point clouds of varying densities. Consequently, it achieves a balanced denoising effect across the overall dataset. The experimental section of this study validates the effectiveness of this method using point clouds collected from real-world outdoor scenes. The results indicate that, compared to traditional methods, the approach proposed in this research not only effectively removes noise but also better preserves the complete environmental features. Overall, this study addresses the issue of point cloud noise removal in real-world outdoor scenes, offering an effective point cloud denoising method as well as new perspectives and tools for point cloud processing and analysis.

## 2. Methodology

### 2.1. Functional Model for Point Cloud Denoising

Point cloud denoising involves analyzing the characteristics and structure of point clouds to eliminate unnecessary noise and enhance the quality and accuracy of the data. Essentially, it is about removing redundant points from a large-scale point set. The functional model for point cloud denoising can be defined as follows [17]:

A noisy point cloud dataset, containing noise points or outliers, can generally be represented as $P=\{p_{i},\;i=1,2,\ldots ,n\}$, which can be approximately represented as

(1) $$p_{i}=q_{i}+e_{i},\;p_{i},q_{i},e_{i}\in R^{3}$$

where $q_{i}$ denotes the real points in the cloud; $e_{i}$ denotes the measurement noise of $q_{i}$. The process of denoising the point cloud can be represented as

(2) $${q_{i}}^{\prime }=ℱ(p_{i})=ℱ(q_{i}+e_{i})$$

where ${q_{i}}^{\prime }$ denotes an approximation of $q_{i}$, and the function $ℱ(p_{i})$ denotes a generalized method for point cloud denoising.

### 2.2. The Overall Framework of the Denoising Method

In real-world outdoor scenes, the randomness and complexity of point cloud noise distribution pose a significant challenge for denoising algorithms. To effectively address this challenge, this study adopts Bayesian estimation to model the point cloud denoising process. Within this framework, the denoising of each point in the point cloud is viewed as a statistical inference problem. The core of this process is to compute the posterior probability of each point being a real point or a noise point. This computation is based on two key components: prior probability and likelihood function. The prior probability reflects the initial assumption about whether a point is a real point or a noise point, while the likelihood function indicates the probability of a point being a real or noise point under the given assumption. The overall framework for point cloud denoising is as follows:

(3) $$P(real|p)=\frac{f_{real}(x,y,z)\times P(real)}{f_{real}(x,y,z)\times P(real)+f_{noise}(x,y,z)\times P(noise)}$$

where the following is true:

- $f_{real}(x,y,z)$ denotes the PDF under the condition that a given point, p, is a real point, i.e., the likelihood function for real points;
- $f_{noise}(x,y,z)$ denotes the PDF under the condition that a given point, p, is a noise point, i.e., the likelihood function for noise points;
- P(real) denotes the prior probability that a given point, p, is a real point;
- P(noise) denotes the prior probability that a given point, p, is a noise point.

In Equation (3), the denominator part denotes the sum of all possibilities of the given point being either a real point or a noise point, serving as a normalization factor for the numerator.

### 2.3. Prior Probabilities of Real Points and Noise Points

When scanning outdoor scenes, LiDAR can achieve high-precision measurements. However, this high precision is affected by the scanning distance. As the scanning distance increases, the laser beam diverges, causing the laser spot size to enlarge with the distance. The increase in spot size not only expands the area covered by the laser spot but also potentially increases the number of interfering objects or noise sources within that area. These interferences or noise sources can scatter, reflect, or absorb the laser, leading to noise mixed into the light signals received from a distance. Furthermore, the enlargement of the laser spot size can also lead to a reduction in the energy density of the spot. A decrease in energy density makes the laser more susceptible to environmental factors during propagation, such as atmospheric scattering and diffraction, thereby increasing the likelihood of noise.

Considering these factors, this study adopts a dynamic adjustment approach to set the prior probabilities of data points in real-world outdoor scenes being real points or noise points. Specifically, the method proposed in this study considers the spatial function relationship between distance and noise probability and dynamically adjusts the prior probability of points based on this relationship. This method aims to reflect the likelihood of noise in distant point cloud, thereby enhancing the accuracy of point cloud denoising. The formula for calculating the prior probability is as follows:

(4) $$P(real)=\max(P_{real}^{base}\times (1-{\left(\frac{d_{i}}{d_{\max}}\right)}^{2}),\;0.7)$$

(5) P(noise)=1−P(real)

where the following is true:

- $P_{real}^{base}$ is the basic prior probability of a point being a real point, where in this case, $P_{real}^{base}=0.95$;
- $d_{i}$ denotes the Euclidean distance from the i point in the point cloud to the center of the LiDAR;
- $d_{\max}$ is the Euclidean distance of the farthest point in the point cloud from the center of the LiDAR, used for normalization to ensure that $\frac{d_{i}}{d_{\max}}$ remains within the range of [0, 1];
- max(⋅,0.7) ensures that the prior probability under the condition that the given point p is a true point is not less than the preset minimum value of 0.7; that is, P(real)>P(noise) is guaranteed in any case.

### 2.4. Likelihood Function of Real Points

In real-world outdoor scenes, point clouds obtained through LiDAR scanning often exhibit specific spatial distribution characteristics. Specifically, point clouds show higher density in areas closer to the center of the LiDAR and relatively sparser distribution in areas farther from the center. This distribution characteristic is due to the variation in point cloud density with the distance from the LiDAR center, which mathematically aligns with the characteristics of a multivariate Gaussian distribution. Hence, this study proposes modeling the PDF of a given point being a real point as a multivariate Gaussian distribution.

The multivariate Gaussian distribution is a commonly used model for describing datasets with multiple variables, effectively reflecting the spatial correlations and density variations in point cloud data. This model considers not only the local characteristics of point cloud data but also the overall statistical features of the data. The advantage of using a multivariate Gaussian distribution as the likelihood function model lies in its mathematical precision and capability to handle complex data. This approach can more accurately estimate the probability of a given point being a real point, thus providing more effective data support during the denoising process. The expression for the PDF of a given point being a real point in a point cloud is as follows:

(6) $$f_{real}(x,y,z)=\frac{1}{{\left(2\pi \right)}^{3/2}\sigma_{x}\sigma_{y}\sigma_{z}}\exp(-\frac{1}{2}[\frac{{\left(x-\mu_{x}\right)}^{2}}{\sigma_{x}{\;}^{2}}+\frac{{\left(y-\mu_{y}\right)}^{2}}{\sigma_{y}{\;}^{2}}+\frac{{\left(z-\mu_{z}\right)}^{2}}{\sigma_{z}{\;}^{2}}])$$

where the following is true:

- $\mu_{x}$, $\mu_{y}$, and $\mu_{z}$ denote the mean of the point cloud coordinates in the X-, Y-, and Z-axis directions, respectively.
- $\sigma_{x}$, $\sigma_{y}$, and $\sigma_{z}$ denote the standard deviation of the point cloud coordinates in the X-, Y-, and Z-axis directions, respectively.

### 2.5. Likelihood Function of Noise Points

In real-world outdoor scenes, the noise distribution in point clouds exhibits significant complexity and randomness. These characteristics pose challenges to conventional parametric modeling methods in capturing these noises. Therefore, this study adopts a non-parametric approach and chooses to use KDE for modeling the PDF under the condition that a given point is a noise point.

KDE is a data-driven, non-parametric technique, advantageous in that it does not rely on prior assumptions about the data distribution. This feature is crucial for dealing with complex or unknown distributions of point cloud noise in real-world outdoor scenes. The flexibility and adaptability of KDE allow it to estimate density functions of any shape adaptively, thus more accurately capturing noise points. The key aspect of KDE is the dynamic calculation of local bandwidth, based on the local density of points. In this way, KDE can better reflect the density variations of point clouds in different ranges, thereby improving the accuracy of noise identification. In this study, the modeling of the PDF for a given point being a noise point in a point cloud is represented using the following formula:

(7) $$f_{noise}(x,y,z)=\frac{1}{{\left(Nh_{i}\right)}^{3}}\sum_{i=1}^{N}K(\frac{x-{\mu_{x}}^{\prime }}{h_{i}},\frac{y-{\mu_{y}}^{\prime }}{h_{i}},\frac{z-{\mu_{z}}^{\prime }}{h_{i}})$$

where the following is true:

- *N* denotes the number of data points in the point cloud;
- K(⋅) denotes the kernel function, and here, K(⋅) is a three-dimensional Gaussian kernel function;
- ${\mu_{x}}^{\prime }$, ${\mu_{y}}^{\prime }$, and ${\mu_{z}}^{\prime }$ denote the local mean of the point cloud in the X-, Y-, and Z-axis directions, respectively, and these values dynamically change with the variation about the point;
- $h_{i}$ denotes the local bandwidth, which determines the width of the kernel function. The appropriate bandwidth is crucial for estimating the PDF. Too small a bandwidth might lead to overfitting of the point cloud noise model, whereas too large a bandwidth could result in an overly smoothed PDF. In this study, the local bandwidth is determined based on Silverman’s rule of thumb. The expression for $h_{i}$ is as follows:

(8) $$h_{i}=h_{global}\cdot {\left(\frac{d_{k}}{N}\right)}^{\frac{1}{D}}$$

(9) $$h_{global}={\left(\frac{4\sigma_{mean}}{3N}\right)}^{\frac{1}{5}}$$

where the following is true:

- $d_{k}$ denotes the Euclidean distance to the *k*th nearest neighbor from the given point;
- D denotes the dimension of the point cloud data;
- $h_{global}$ denotes the global bandwidth;
- $\sigma_{mean}$ denotes the mean standard deviation of the point cloud in the X-, Y-, and Z-axis directions.

## 3. Experiment

### 3.1. Experimental Setup

#### 3.1.1. LiDAR Parameters and Dataset

**Figure 1:**
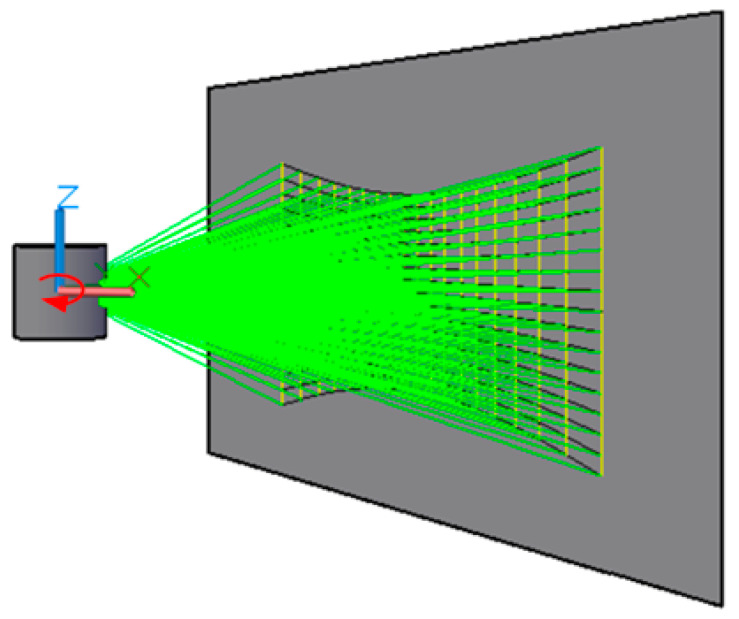
Diagram of scanning principle of LiDAR (16 lines).

In this study, the point cloud dataset was collected in real-world outdoor scenes using the VanJee WLR-720 LiDAR, the instrument is manufactured by VanJee Technology, located in Beijing, China. The scanning principle of the LiDAR and its schematic are shown in Figure 1. The WLR-720 LiDAR features 16 scanning beams with an effective ranging capability of 120 m (0.5 m~70 m @10% reflectivity). This device can perform 360° omnidirectional scanning with a scanning frequency of up to 20 Hz. Vertically, it has a field of view of 30° (ranging from −16° to 14°), enabling it to cover a wide vertical space. In terms of resolution, the WLR-720 offers 0.1° horizontally and 2° vertically, with a ranging accuracy of ±2 cm (typical).

**Figure 2:**
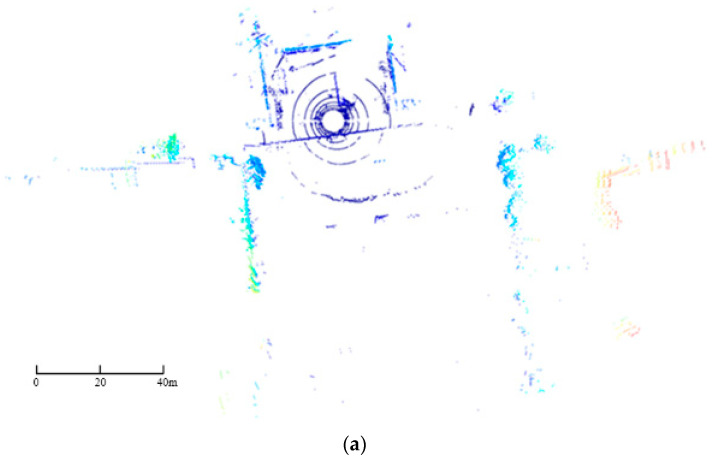

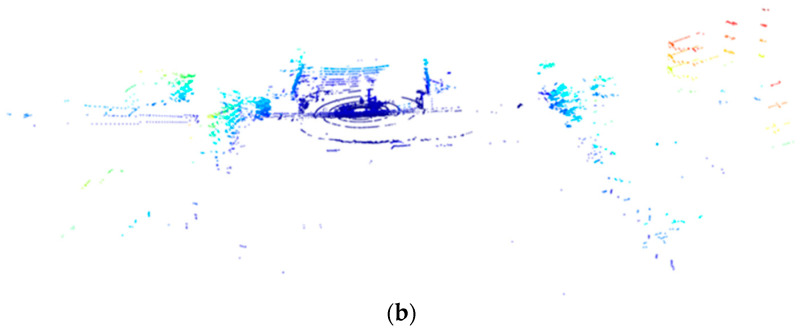
Raw point cloud in real-world outdoor scenes. (**a**) Plan view. (**b**) Side view.

In the experiment, the LiDAR was used to collect a total of 303,965 data points in an outdoor real environment. The obtained point cloud not only includes the three-dimensional spatial coordinates of a large number of data points but also the reflectivity of the targets. The scanned objects primarily included buildings, trees, the ground, vehicles, and pedestrians. The collected dataset was stored in the CSV format. To ensure data consistency and usability, the raw point cloud underwent preprocessing, including the removal of header rows and data type conversion. The raw point cloud is shown in Figure 2.

#### 3.1.2. Experimental Environment and Tools

To validate the effectiveness of the point cloud denoising method proposed in this study, corresponding experiments were conducted in specific hardware and software environments. The configuration of the computing platform used in the experiment is as follows:

(1) Hardware environment: The platform is equipped with a high-performance Intel^®^ Core™ i9-14900 CPU, produced by Intel Corporation, located in Santa Clara, CA, USA, the CPU has a clock speed of up to 5.8 GHz, ensuring high efficiency in data processing. For graphics processing, the platform includes an NVIDIA^®^ GeForce RTXTM 4060 graphics card, provided by NVIDIA Corporation, which is based in Santa Clara, CA, USA as well, offering robust graphical processing capabilities. Additionally, the system has Samsung DDR5 32 GB memory, sourced from Samsung Electronics, headquartered in Suwon-si, Gyeonggi-do, South Korea, ensuring ample space for data processing and storage.

(2) Software environment: The experiments were run on the Windows 10 operating system. Data processing and analysis mainly relied on the Python 3.8.8 environment, along with professional scientific computing libraries. These libraries include Spyder 5.4.3 as the integrated development environment, pandas 1.4.3 and NumPy 1.22.0 for data processing, SciPy 1.7.3 for scientific computing, and Open3D 0.13.0 for the visualization and processing of the point cloud dataset.

### 3.2. Experimental Result

#### 3.2.1. Spatial Distribution of Points in Different Ranges

**Figure 3:**
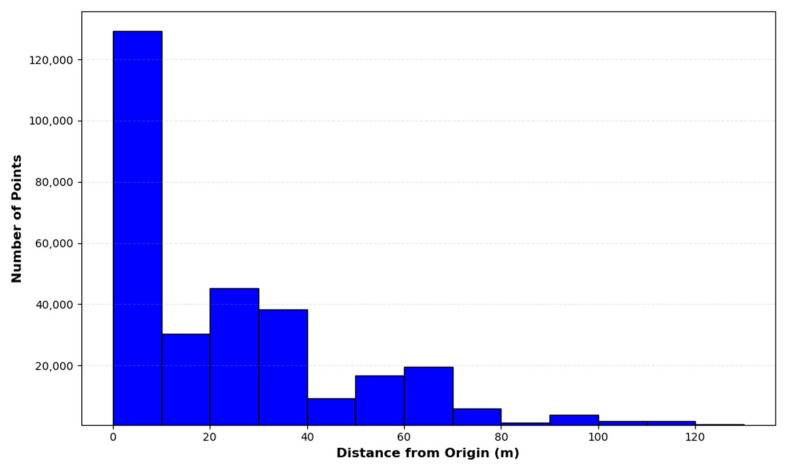
Spatial distribution of data points in different ranges.

To understand the spatial distribution characteristics of the raw point cloud, this experiment plotted a histogram showing the distribution of points relative to the center of the LiDAR, as depicted in Figure 3. In this histogram, the horizontal axis represents the distance of data points in the point cloud from the center of the LiDAR, divided into intervals of 10 m; the vertical axis indicates the number of points within each interval.

The histogram clearly reveals an interesting phenomenon: the density of the point cloud decreases with increasing distance, aligning with the inherent characteristics of LiDAR measurements. In particular, within 10 m of the LiDAR center, the number of points reaches its maximum, possibly reflecting the LiDAR’s high resolution and excellent detection capability for close-range targets. As the distance increases, the number of points decreases, mainly related to the sparse distribution of objects within the scanning area. In further distant intervals, the significant reduction in the number of points is associated with physical factors such as the maximum effective detection range of the device, laser beam divergence, atmospheric scattering, and reduced reflectivity of target surfaces. The histogram provides an intuitive understanding of the LiDAR’s detection capabilities and the characteristics of the scanning area, which is crucial for further processing and analyzing point clouds in real-world outdoor scenes.

#### 3.2.2. Dynamic Prior Probabilities of Real Points and Noise Points

**Figure 4:**
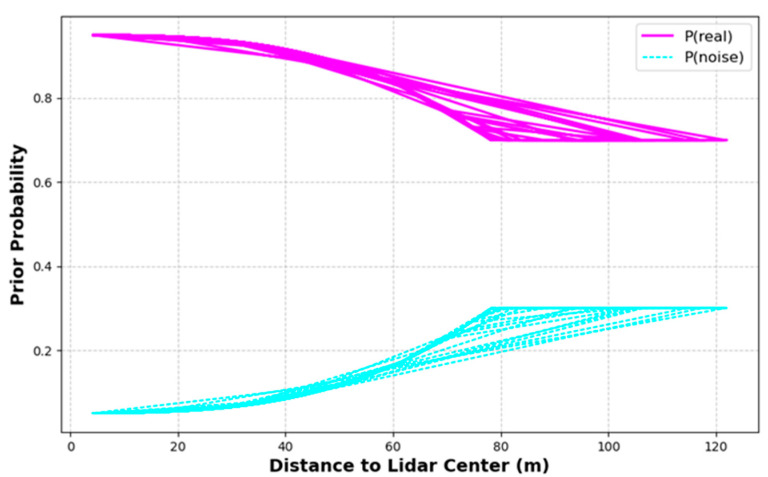
Dynamic prior probabilities of data points in the point cloud relative to the distance from the LiDAR center.

To address the randomness and complexity of point cloud noise distribution in real-world outdoor scenarios, this study adopts a dynamic approach to set the prior probabilities for each point in the point cloud to be either a real point or a noise point under given conditions. Based on Equations (4) and (5), the prior probabilities for each point in the raw point cloud were calculated, with the results shown in Figure 4.

In the graphical representation, the pink line indicates the prior probability of real points, and the light turquoise line represents the prior probability of noise points, with the distance measured relative to the center of the LiDAR. As observed in Figure 4, the prior probability of real points gradually decreases with increasing distance, while the prior probability of noise points correspondingly rises. This probability distribution reflects that in ranges farther from the LiDAR center, the likelihood of data points being noise points increases, and the likelihood of them being real points decreases.

The dynamic setting strategy of prior probabilities offers a new perspective for point cloud denoising in real-world outdoor scenes. It considers the functional relationship between the distance of points from the LiDAR center, emphasizing the spatial dependency that needs to be considered in the denoising process; i.e., the signal-to-noise ratio of points in different spatial ranges may vary significantly. The advantage of this strategy lies in its ability to adapt to the actual distribution characteristics of the point cloud, providing a realistic method of setting prior probabilities, especially suitable for point clouds collected in real-world outdoor scenes.

#### 3.2.3. Multivariate Gaussian Distribution Surface Fitting for the Likelihood Values of Real Points

**Figure 5:**
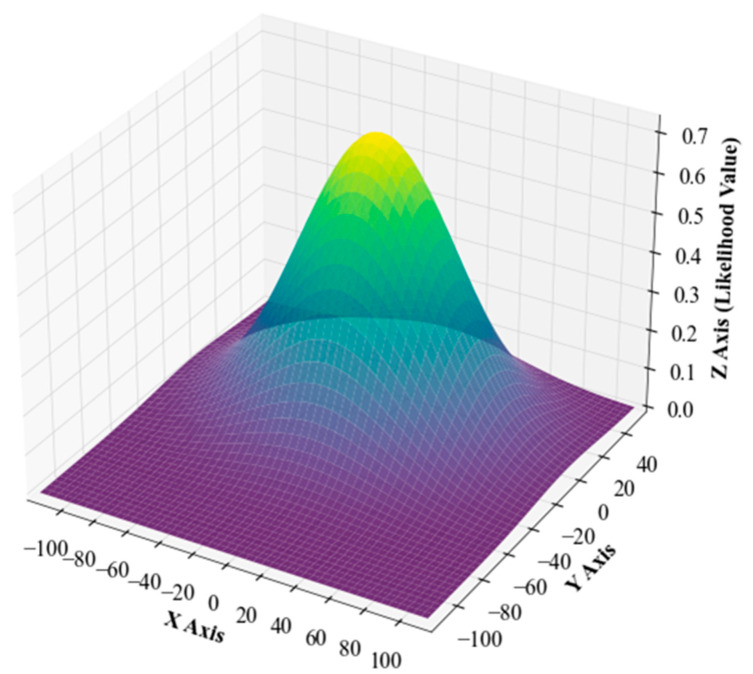
Multivariate Gaussian surface fitting of the likelihood values of real points in real-world outdoor scenes.

To validate the assumption that the PDF of real points in outdoor scenes conforms to a multivariate Gaussian distribution, this study first applied traditional denoising techniques to the collected raw point cloud. Subsequently, the likelihood values for each point in the denoised point cloud were calculated using Equation (6). Finally, a multivariate Gaussian distribution surface fitting was performed on the calculated likelihood values for each point, with the results shown in Figure 5.

The likelihood values exhibit a distinct trend: the peak is primarily concentrated near the coordinate origin and decreases with increasing distance from the LiDAR center. This characteristic aligns with the expected behavior of a multivariate Gaussian distribution and provides strong evidence for the hypothesis that the PDF of real points in outdoor environments can be well approximated by a multivariate Gaussian distribution. The shape of this PDF provides a theoretical basis for point cloud denoising methods in outdoor scenes. By applying this distribution in point cloud denoising, real points can be more accurately distinguished. Additionally, this surface fitting approach can also help to assess the potential spatial variability and complexity in point cloud datasets.

#### 3.2.4. Impact of the Number of Neighborhood Points on the Local Bandwidth

**Figure 6:**
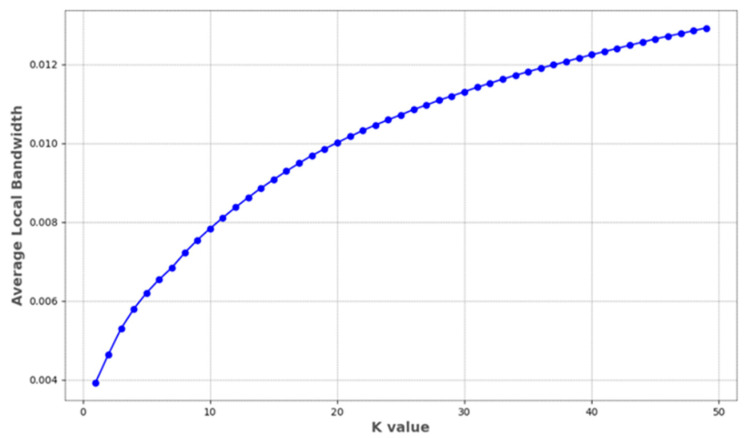
Impact of the number of neighborhood points on the average local bandwidth.

In this study, the local bandwidth plays a crucial role in noise removal, but it is controlled by the number of neighboring points and dynamically adjusted with changes in the points. To gain a comprehensive understanding of how variations in the number of neighborhood points affect local bandwidth, an analysis was conducted on the overall impact of the neighborhood point count on the average local bandwidth from a holistic point cloud perspective. The experimental results are illustrated in Figure 6.

The average local bandwidth increases with the number of neighborhood points, a trend particularly pronounced in the range of smaller neighborhood point counts (K values). The rapid increase in local bandwidth at small scales reveals the point cloud’s high sensitivity to small-scale changes. This sensitivity means that in KDE, each point has a strong interaction with its neighboring points. However, this could also lead to the over-amplification of noise, adversely affecting the overall stability of the density estimation. As the number of neighborhood points increases, the rate of increase in the average local bandwidth starts to slow down. This indicates that density estimates tend to stabilize over larger neighborhood ranges, reflecting the integration of information from a broader area. The result of this information integration is an increased smoothness of the density estimate, helping to mitigate the impact of noise, though it may somewhat obscure important local variations in the data to a certain extent. Once the number of neighborhood points reaches a certain level, the increase in the average local bandwidth tends to saturate. This suggests that further increases in the number of neighborhood points have limited contributions to improving local density estimation, indicating that the characteristic scale of the data’s intrinsic structure has been reached. Continuing to increase the number of neighborhood points will not significantly affect the outcome of the density estimation.

#### 3.2.5. The Impact of Dynamic Prior Probability and Local Bandwidth on Noise Removal

**Figure 7:**
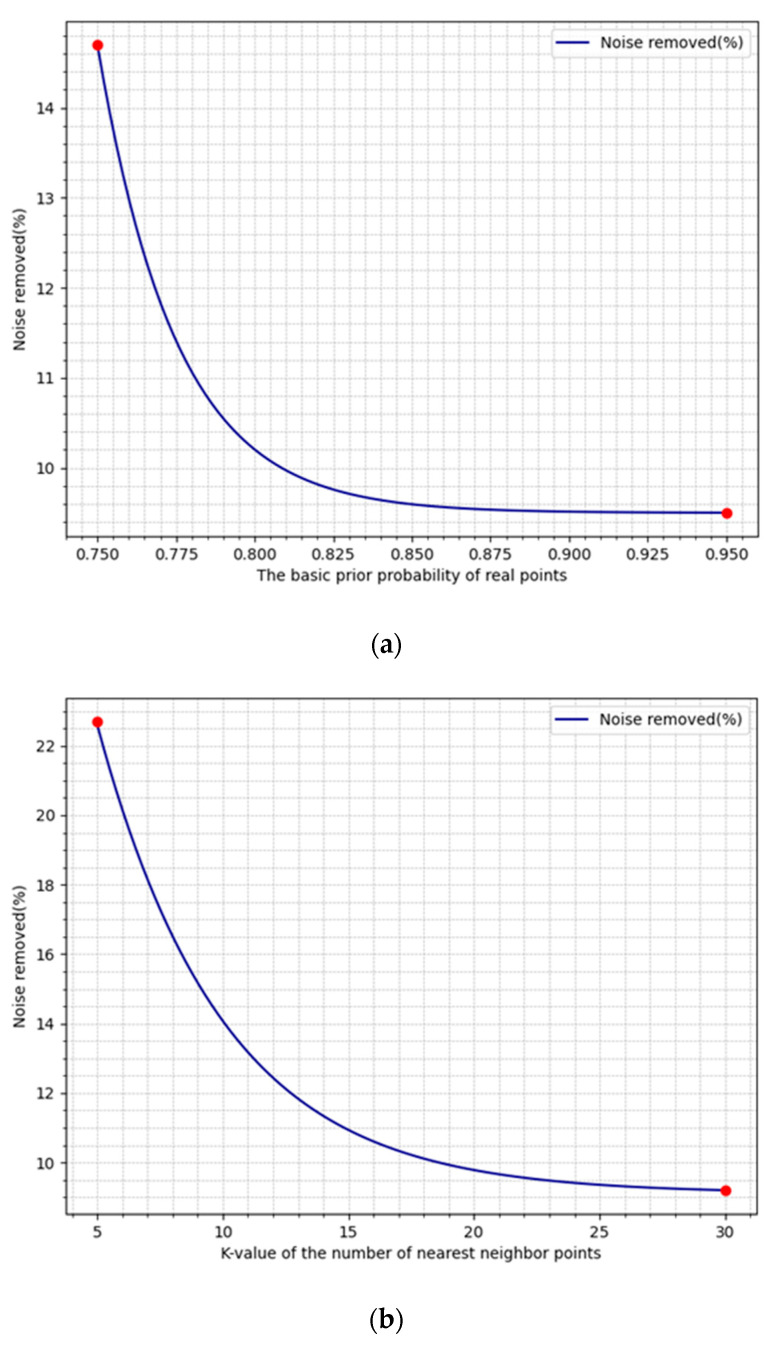
The impact of prior probability and the number of neighboring points (K) on noise removal. (**a**) The impact of prior probability changes on noise removal. (**b**) The impact of variations in the number of neighboring points (K) on noise removal.

Dynamic prior probabilities and local bandwidth play crucial roles in point cloud noise removal. As known from Equations (4) and (5), the prior probabilities of real points and noise points are controlled by the basic prior probability of real points, while Equation (8) reveals that the local bandwidth is primarily controlled by the number of neighboring points (K). Therefore, the experiment analyzed the effects of variations in these two parameters on noise removal, with the results illustrated in Figure 7.

Figure 7a indicates that when the basic prior probability of real points is in the range of 0.75–0.80, the variation in noise removal is quite significant. This suggests that assuming a lower basic prior probability of real points can lead to more aggressive noise removal. However, as the value increases from 0.80 to 0.95, the changes in the basic prior probability of real points contribute less to noise removal. Figure 7b shows that when the number of nearest neighbor points (K) is between 5 and 15, the percentage of noise removed is higher. This is because a smaller number of neighboring points allows the algorithm to identify noise more finely on a local scale, but too small a K value may lead to excessive smoothing of the point cloud. As K increases, noise removal gradually stabilizes. Figure 7 provides an intuitive understanding of how the basic prior probability of real points and the number of nearest neighbor points (K) affect noise removal.

#### 3.2.6. Point Cloud Denoising in Real-World Outdoor Scenes Based on the Overall Framework

**Figure 8:**
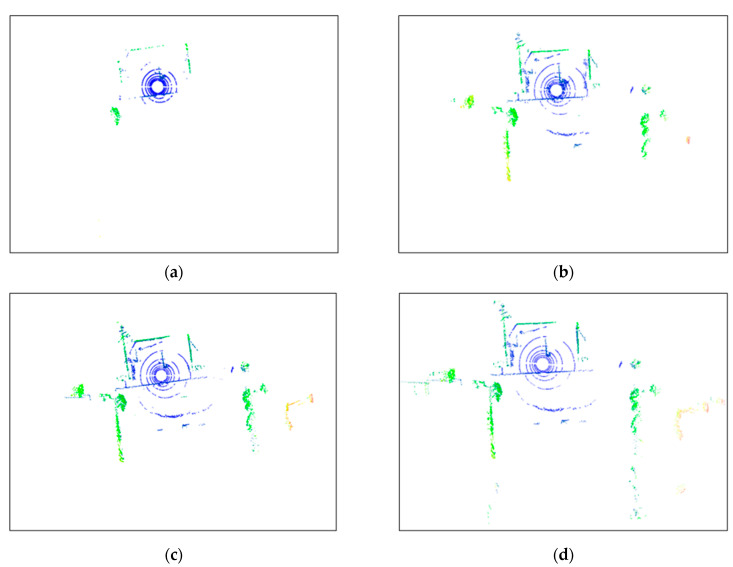
Comparing performance of ROR, SOR, DROR, and AKA-LDGS filter (plan view). (**a**) ROR filter, (**b**) SOR filter, (**c**) DROR filter, (**d**) AKA-LDGS filter.

**Figure 9:**
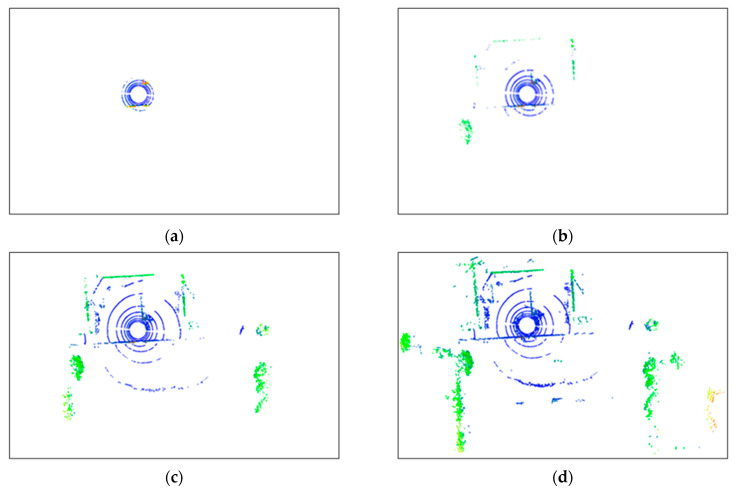
Performance of the ROR filter with different search radius (plan view). (**a**) *SR* = 0.03 m, (**b**) *SR* = 0.05 m, (**c**) *SR* = 0.10 m, (**d**) *SR* = 0.50 m.

**Figure 10:**
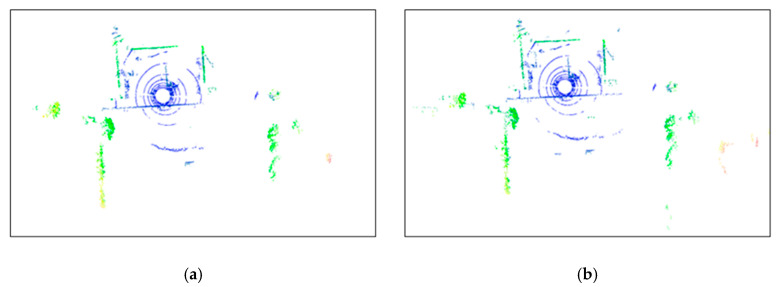
Performance of the SOR filter with different standard deviation ratio (plan view). (**a**) *SD_ratio_* = 1, (**b**) *SD_ratio_* = 2.

To evaluate the effectiveness of the proposed method in denoising point clouds in real-world outdoor scenes, a denoising experiment was conducted on the raw point cloud data collected from such scenes using Equation (3). This method was compared and analyzed against classic radius-based and statistics-based filtering methods. Figure 8 shows the planar views of LiDAR scans before and after applying the ROR (Radius Outlier Removal), SOR (Statistical Outlier Removal), DROR (Dynamic Radius Outlier Removal) [34], and AKA-LDGS filters. To enhance visualization, points within 2 m of the Lidar center were removed using a distance threshold. Figure 8a, Figure 8b, Figure 8c and Figure 8d, respectively, show the point clouds after denoising using the ROR, SOR, DROR, and AKA-LDGS filters applied to the raw point cloud. While all four methods effectively denoise the raw point cloud, the AKA-LDGS filter manages to preserve more environmental features during the denoising process. Figure 9 shows the trade-off between noise removal and preserving environmental features with the variation in the search radius in the ROR filter. Figure 10 shows the trade-off achieved by the SOR filter between noise removal and environmental feature preservation as the standard deviation multiplier changes.

**Figure 11:**
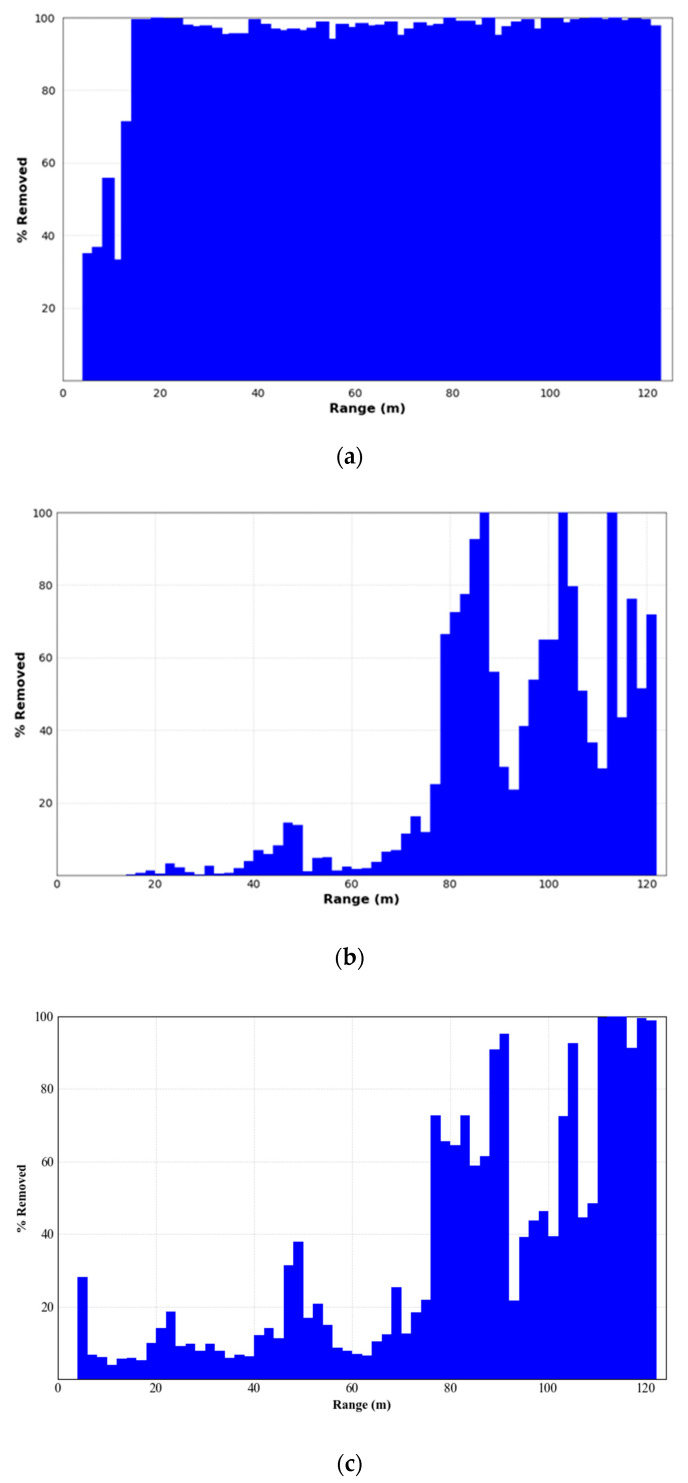

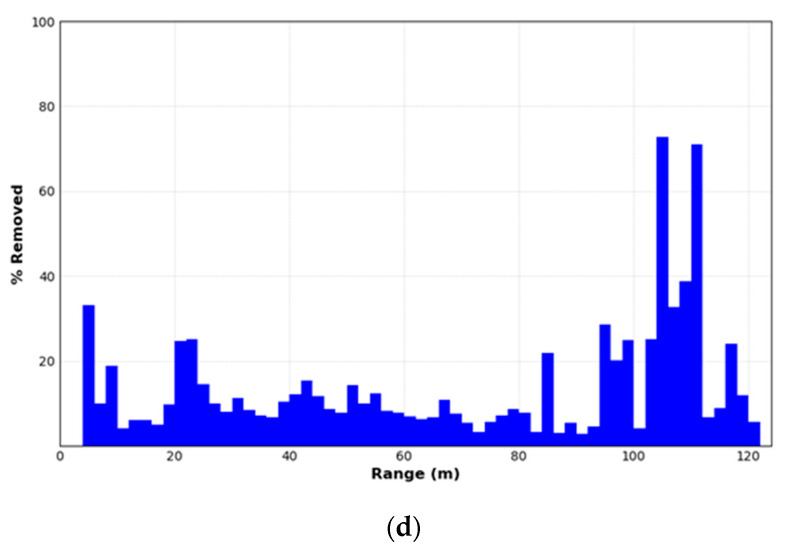
The average performance of points removed per range bin. (**a**) ROR, (**b**) SOR, (**c**) DROR, (**d**) AKA-LDGS.

To quantify the quality of environmental feature preservation by the four filters, a plot was created showing the percentage of noise removed relative to the range, with the results shown in Figure 11.

The ROR filter exhibited near 100% removal of noise points beyond approximately 20 m from the center of the LiDAR. However, as shown in Figure 3, there are still a considerable number of points distributed in the range of 20 to 80 m from the LiDAR center, but almost all environmental features within this range were removed. Therefore, when using the ROR filter for noise removal, careful parameter adjustment is necessary to balance the preservation of environmental features. The SOR filter showed less than 20% removal within approximately 80 m of the LiDAR center, with three peaks of near 100% noise removal beyond this range, due to the extreme sparsity of the point cloud in these areas. The SOR filter’s performance in preserving environmental features decreases with increasing distance from the LiDAR center. The DROR filter achieved relatively balanced noise removal within approximately 75 m of the center of the LiDAR. However, beyond the range, a higher proportion of point clouds were removed as noise, resulting in some degree of degradation of environmental features. The AKA-LDGS filter maintained a relatively balanced noise removal across the LiDAR’s ranging scope, preserving the overall environmental features of the point cloud well. Beyond approximately 95 m from the LiDAR center, there was a relatively higher rate of noise removal, attributable to fewer targets, greater measuring distances, and environmental interferences in this range. This result demonstrates the superiority of the AKA-LDGS filter in removing point cloud noise in real-world outdoor scenes.

#### 3.2.7. Point Cloud Denoising Effectiveness Assessment

**Figure 12:**
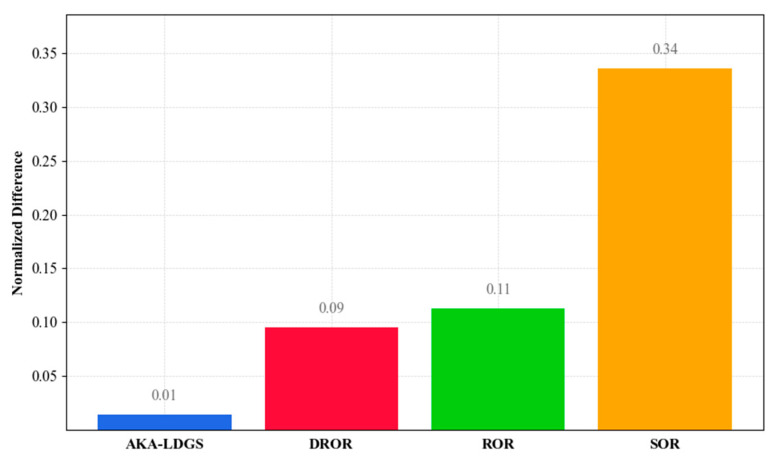
Comparison of denoising effects between the AKA-LDGS, DROR, ROR, and SOR filters.

In the evaluation of noise removal in real-world outdoor scenes, there is the challenge of obtaining actual point cloud datasets. To fairly compare the denoising method proposed in this study with other commonly used denoising methods, a relative evaluation approach was employed to quantify the effects of different denoising techniques, introducing normalized median variance as an assessment metric. This method takes each point in the denoised point cloud as a reference, searches for neighboring points in the raw point cloud, and calculates the variance of these neighboring points, reflecting the consistency and stability of point distribution within local areas. In the noise removal evaluation, the use of the median variance instead of the mean reduces the impact of outliers on the evaluation results. Subsequently, a normalization of the median variance was carried out to ensure that the evaluation results were not influenced by data scale, thereby providing consistency and fairness in the comparison among different denoising methods. The denoising performance of the four filters is shown in Figure 12.

Figure 12 provides a quantitative comparison of the performance of the four denoising filters, assessed through the difference in normalized median variance. A smaller normalized value indicates that the denoised point cloud retains a higher similarity in local spatial consistency with the raw point cloud. As shown in Figure 12, the AKA-LDGS filter exhibits the smallest normalized difference, indicating its most significant denoising effect in real-world outdoor scenes. In comparison, the DROR filter performs second best, the ROR filter ranks medium, while the SOR filter exhibits the greatest normalized difference. Combined with the visual assessment in Figure 8d, it can be further confirmed that the AKA-LDGS filter demonstrates better denoising performance while preserving the environmental features of outdoor real-world scene point clouds.

## 4. Discussion

### 4.1. Application of Dynamic Prior and Adaptive KDE in Point Cloud Denoising

This study introduces a noise removal method that combines dynamic prior probability adjustment with local bandwidth tuning, demonstrating superior performance in processing large-scale 3D point cloud datasets. Traditional denoising methods often rely on fixed thresholds or parameters [29], limiting their adaptability when dealing with point clouds of varying densities and noise levels. In contrast, the method presented in this paper dynamically adjusts the prior probability of each point, combined with bandwidth adjustments based on local density, allowing the algorithm to better adapt to the local characteristics of the data. This is particularly important when dealing with complex scenarios, such as scans of buildings or urban environments, where the complexity of these environments often results in significant differences in point cloud density and noise levels across different areas [33,35]. Another advantage of this method is its robustness in parameter selection. Although the number of neighboring points needs to be predetermined, the sensitivity of the model to this parameter is relatively low due to the dynamic adjustments of prior probability and local bandwidth in subsequent computations. This enables the method to achieve good denoising results without extensive parameter tuning.

### 4.2. Limitations and Future Directions for Optimization of the Method

While the method proposed in this study demonstrates certain advantages in noise removal, there are still some limitations. Firstly, although the construction and querying process of KD-Tree is more efficient than conventional neighborhood search methods, dealing with extremely large datasets still poses challenges in terms of time and space complexity. Especially in environments with limited memory resources, the processing of large-scale point clouds may be constrained [36]. Moreover, while the dynamically adjusted prior probabilities provide a mechanism to adjust noise estimation based on the spatial distribution of points, this approach still relies on assumptions about spatial distribution. In some extreme cases, such as scenarios where noise and signal points highly overlap in space, these assumptions may lead to a decrease in denoising effectiveness. Future research could improve in two directions: one is to develop more efficient data structures and algorithms to further reduce computational and storage requirements, such as by re-expressing the point cloud data structure using knowledge from the complex domain that we are currently exploring in depth; the second is to explore the integration of other types of prior knowledge to improve the accuracy of noise estimation, for example, learning the structural features of point clouds through deep learning [18].

### 4.3. Theoretical Significance and Technological Prospects of the Overall Framework

This study offers a novel and effective perspective in the field of point cloud denoising. Theoretically, the method combines spatial statistics and probabilistic models, providing a new way to understand and process point clouds. By considering the spatial structure and distribution characteristics of point clouds, this method offers a more refined strategy for noise removal and provides new ideas and frameworks for subsequent related research, especially when dealing with complex or irregular point clouds. On the application level, with the rapid development of mobile mapping and autonomous driving technologies, the efficient processing of point clouds is becoming increasingly important [37]. This method demonstrates potential in noise removal in real-world outdoor scenarios, which is crucial for the practical application of point cloud data in these rapidly advancing fields.

## 5. Conclusions

This study introduces a point cloud denoising method for real-world outdoor scenes, combining spatial statistical analysis and probabilistic modeling. By utilizing an efficient spatial search implemented through KD-Tree, dynamically adjusting prior probabilities based on local characteristics of points and adopting adaptive KDE to fit the complex spatial structures of point clouds, this method demonstrates outstanding performance in noise removal in real-world outdoor scenes. The dynamic adjustment strategy for prior probabilities and the use of adaptive KDE in the likelihood function of noise points provide enhanced adaptability for noise removal, which can effectively distinguish between real points and noise points based on the spatial distribution characteristics of point clouds, thereby achieving efficient point cloud denoising. Additionally, by comparing the point cloud before and after denoising, this method is confirmed to not only improve the signal-to-noise ratio of the data but also to preserve critical geometric feature information, which is vital for subsequent point cloud analysis and applications. Overall, the method proposed in this study offers new perspectives and tools for noise removal in real-world outdoor scenes.

## Data Availability

The datasets generated and analyzed during the current study are not publicly available due to other considerations and constraints specific to the nature of the research. However, the datasets are available from the corresponding author upon reasonable request.
